# 

**Published:** 1871-06

**Authors:** 


PUBLISHED MONTHLY, AT $3 00 PER ANNUM IN ADVANCE.

### ATLANTA

### Medical and Surgical Journal.

EDITED BY

W. F. WESTMORELAND, M. D.,

*Prof. of the Principles and Practice of Surgery in the Atlanta Medical College.*

AND

J. G. WESTMORELAND, M. D.,

*Prof. of Materia Medica and Therapeutics in Atlanta Medical College.*

Pax et Scientia, sed veritas sine timore.

VOL. IXB JUNE, 1871. No. 3.

ATLANTA, GEORGIA:

THE TRUE GEORGIAN BOOK AND JOB PRINTING OFFICE.

1871.

EACH NUMBER CONTAINS SIXTY-FOUR PAGES.

